# Primary malignant epithelioid hemangioendothelioma of the pleura: A review and report of a novel case

**DOI:** 10.1002/ccr3.6211

**Published:** 2022-08-11

**Authors:** Alireza Rezvani, Reza Shahriarirad, Amirhossein Erfani, Keivan Ranjbar

**Affiliations:** ^1^ Bone Marrow Transplantation Center, Nemazi Hospital Shiraz University of Medical Sciences Shiraz Iran; ^2^ Thoracic and Vascular Surgery Research Center Shiraz University of Medical Science Shiraz Iran; ^3^ Student Research Committee Shiraz University of Medical Sciences Shiraz Iran

**Keywords:** case report, epithelioid hemangioendothelioma, pleura, review

## Abstract

Epithelioid hemangioendothelioma is considered an uncommon tumor originating from vascular tissues. Although this disease is an extremely rare malignant cancer, its pleural subtype is even less common. We discuss a 68‐year‐old man with isolated pleural epithelioid hemangioendothelioma, along with a literature review of all similar cases.

## Graphical abstract

Primary malignantepithelioid hemangioendothelioma of the pleura in a 68‐year‐old male. A hypermetabolic nodule in the right lower lobe, adjacent to oblique right fissure with SUV of 4.39 at maximum and hypermetabolic primary tumoral involvement in the right parietal and mediastinal pleura, along with malignant right effusion were noted. Diffuse irregular pleural thickening is noted on the Right side, showing increased FDG activity (SUVmax: up to 7.98).

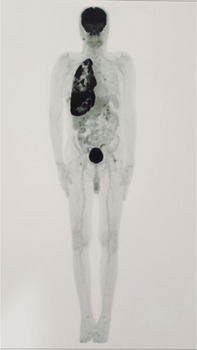

## 1 INTRODUCTION

Epithelioid hemangioendothelioma (EH) is considered an uncommon tumor originating from vascular tissue with intermediate behavior of malignancy originating from various sites such as the brain, meninges, bone, gastrointestinal tract, liver, skin, breast, testis, and mediastinum.^1^, ^2^, ^3^ Although this tumor can affect various tissues, EH originating from the pleural is less described and believed to act more aggressively in its clinical course.^1^, ^4^ Here, we report a case of primary malignant EH of the pleura and a literature review.

## 2 CASE PRESENTATION

A 68‐year‐old man was referred to our clinic for pleuritic chest pain and weight loss (about 3 kg in 4 months). He was referred to our hematology clinic, located in Shiraz, Iran, and affiliated with Shiraz University of Medical Sciences. The patient had no past medical history of smoking or exposure to asbestos. Physical examination, laboratory findings, and abdominal pelvic sonography were not decisive for diagnosis. Chest x‐ray (CXR) revealed a thick wall cavity on the right side.

**FIGURE 1:**
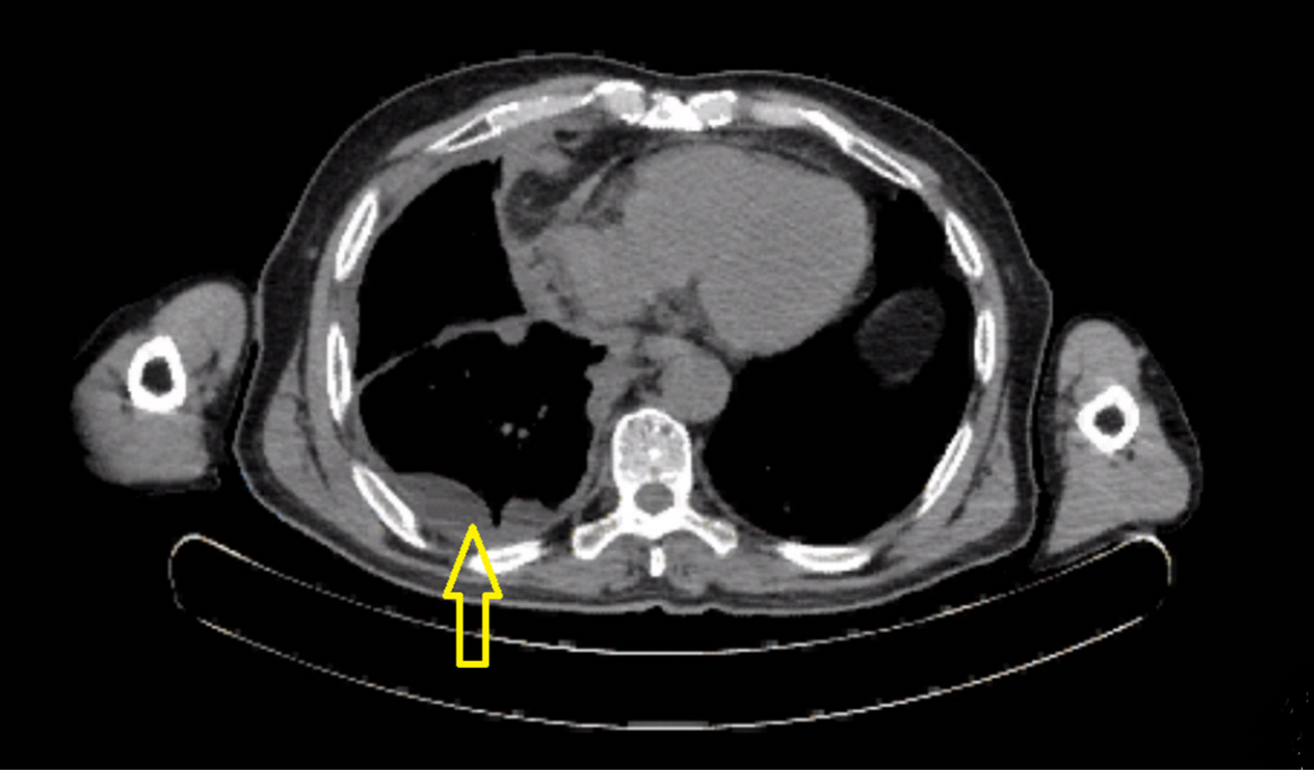
Chest computerized tomography in favor of left‐sided pleural effusion and mass (demonstrated with yellow arrow)

A chest computerized tomography (CT) revealed two calcified nodules on the left side of the chest, including a 5.5 mm‐sized and a 4 mm‐sized in the left lung apex. There was an enhanced irregular thickening of the right parietal pleura with severe pleural effusion and the passive collapse of the right lower lobe and parts of the right upper and middle lobe (Figure 1).

The patient underwent diagnostic thoracentesis that showed a total count: of 540/μl, Red blood cell (RBC): 380/μl, Neutrophil 2/μl, Lymphocyte 158/μl, Sugar fluid: 125 mg/dl, Protein fluid 4.2 gr/dl, Lactate dehydrogenase (LDH) 323 IU/L Adenosine deaminase (ADA) fluid: 5.0 U/L (all in the normal range) and negative bacteriology. Also, right pleural fluid tap cytology was negative for malignant cells.

**FIGURE 2:**
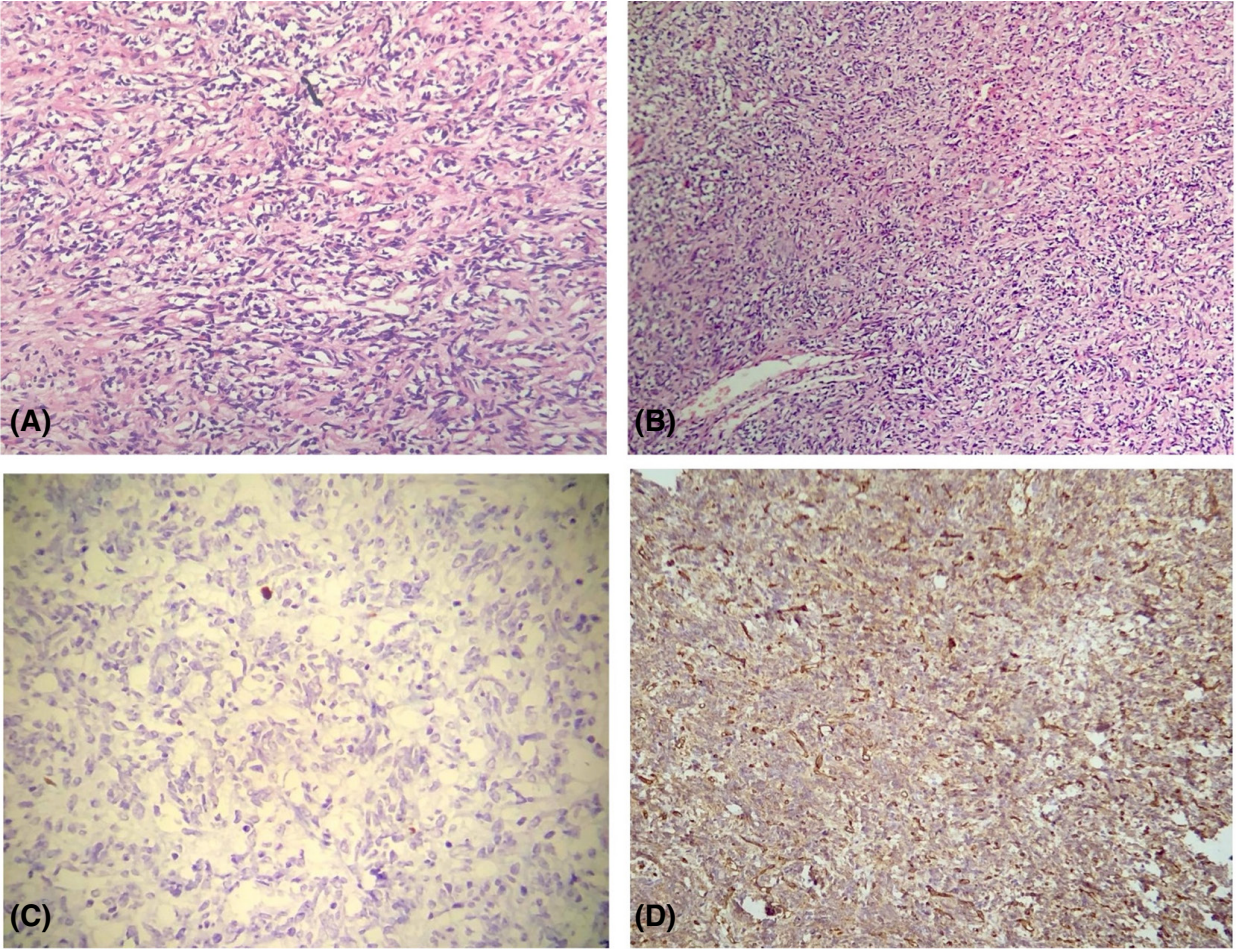
Pathological evaluation of primary malignant epithelioid hemangioendothelioma of the pleura; (A) section of tumor which shows a spindle cell proliferation without nuclear atypia (H&E, ×200); (B) spindle cell proliferation with hemangiopericytoma like vessels (H&E, ×100); (C) Ki67 staining which shows a low proliferative index; (D) positive CD34 immunostaining

The patient underwent thoracoscopy, and a biopsy of the pleura was taken. Grossly, the specimen consisted of a resected lung tissue measuring 4.0 × 3.0 × 2.0 cm in maximal dimension. Microscopic description showed numerous ill‐defined and coalescing nodules composed of hypercellular epithelioid cell proliferation with uniform nuclei and prominent nuclei, showing frequent intracytoplasmic vacuoles. The tumors were infiltrative and showed desmoplastic fibromyxoid stroma. The cytological atypia was overall mild with very focal pleomorphism, but the hypercellularity was prominent, necrosis was abundant, and the mitotic rate was highly elevated (>30/10 hpd). The tumor cells were variably positive for AE1.3, CAM 5.2, CD31, and ERG, while negative for CD34, MUC4, MOC31, BerEp4, TTF1, calretinin, P40, desmin, S100, and Melan‐A. KI69 index was elevated (70%). According to the above findings and the immunohistology evaluation, the diagnosis of pleural EHE was made. (Figure 2).

**FIGURE 3:**
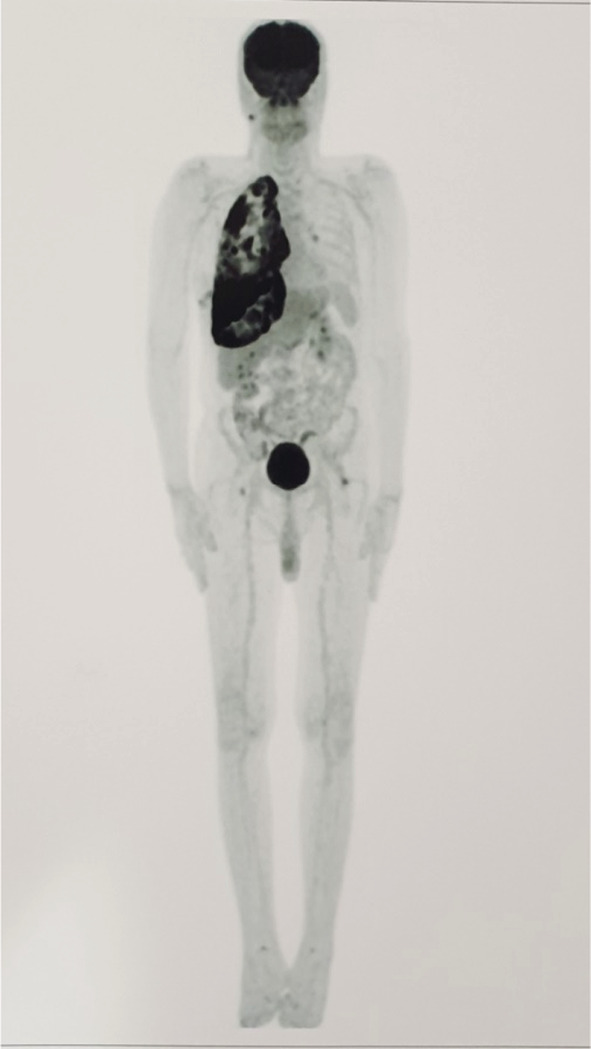
A hypermetabolic nodule in the right lower lobe, adjacent to oblique right fissure with SUV of 4.39 at maximum and hypermetabolic primary tumoral involvement in the right parietal and mediastinal pleura, along with malignant right effusion were noted. Diffuse irregular pleural thickening is noted on the right side, showing increased FDG activity (SUVmax: up to 7.98)

Analyte‐specific reagents (ASR) disclaimer on the positive and negative controls stain appropriately. The patient was diagnosed with pleural EHE following the examination and laboratory results. So, a fluorodeoxyglucose positron emission tomography (FDG‐PET) scan was performed to evaluate the staging and metastasis of epithelioid hemangioendothelioma. The PET scan revealed two non‐FDG‐avid calcified nodules in the left upper lobe, a diffuse irregular pleural thickening on the right side that showed increased FDG activity with a standardized uptake value^5^ 7.98 at maximum, evidence of right pleural effusion with increased FDG uptake. No significant uptakes in other known nodules were noted, and no metastatic lesions were found (Figure 3).

Due to no response to Pazopanib (800 mg once daily) for 4 months, paclitaxel (175 mg/m^2^) was started, and the patient was scheduled for pleurectomy; however, he did not consent and was lost to follow‐up.

## 3 DISCUSSION AND CONCLUSION

**FIGURE 4:**
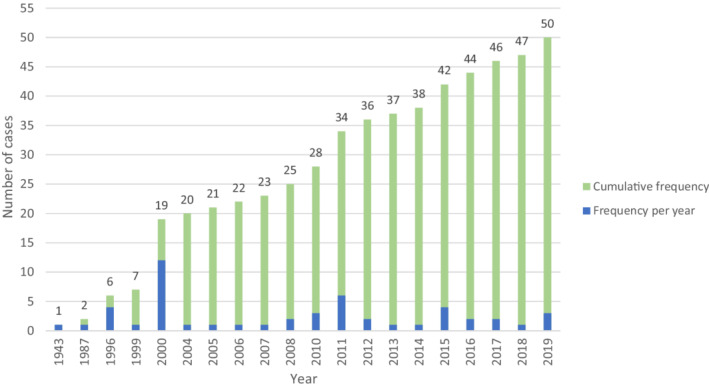
Frequency of primary pleural malignant epithelioid hemangioendothelioma from 1943 to 2019 based on published research

Although EHE is considered an extremely rare malignant cancer, and its pleural subtype is even less common, further characterizing its clinical course and treatment outcomes is necessary. Therefore, a thorough literature review was conducted. We recorded data of 49 patients from reports published in PubMed, Scholar, Medline, and Scopus using the terms “intravascular bronchoalveolar tumor” or “pleural epithelioid hemangioendothelioma” or a combination of both. Cases of pulmonary EHE with metastasis to the pleura and cases in which the histologic diagnosis was epithelioid angiosarcoma were excluded; however, distinguishing the tumors' primary site in pleural and lung involvement was not possible. Therefore, extracting the exact number of cases in the literature review is not possible.^1^, ^2^, ^3^, ^4^, ^5^, ^6^, ^7^, ^8^, ^9^, ^10^, ^11^, ^12^, ^13^, ^14^, ^15^, ^16^, ^17^, ^18^, ^19^, ^20^, ^21^, ^22^, ^23^, ^24^, ^25^, ^26^, ^27^, ^28^, ^29^, ^30^, ^31^, ^32^, ^33^, ^34^, ^35^ Since there was no available national or international database regarding EHE cases, we created a database based on all available reports in the literature. Additionally, we added our case of pleural EHE to this database, resulting in a total of 50 primary cases of pleural EHE. Figure 4 demonstrates the frequency of reported primary pleural malignant epithelioid hemangioendothelioma until now based on published research. Also, Table 1 demonstrates the clinical, demographical, and histological features of primary pleural hemangioendothelioma based on a review of published literature.

**TABLE 1:** Clinical, demographical, and histological features of primary pleural hemangioendothelioma based on a review of published literature

No of patients	Authors/reference	Age/sex	Symptoms (no.)	Asbestos exposure/cigarette	Chest radiography (no.)	Factors	Other involved sites	Treatment	Survival (months)
1	Stout et al.^6^	61/M	Left lower chest pain, night sweats, weakness, hemoptysis	NA	NA (thickening of pleura in autopsy)	NA	Hilar lymph node, left lung, diaphragm	Thoracentesis	8
2	Yousem et al.^18^	34/M	Dyspnea	NA	Bilateral pleural effusion	NA	None	None	3
3	Lin et al.^19^	51/M	NA	Asbestos (+), cigarette (+)	Pleural thickening and effusion	Vimentin (+), Keratin (−), CD31(+), CD34 (+), UEA‐1 (+), VWF (+), Collagen IV (+)	None	Pleurectomy	NA
4		58/M	NA	NA	Pleural thickening	Vimentin (+), CD34 (−), UEA‐1 (+), VWF (−), Collagen IV (+)	None	Pleurectomy	6
5		56/M	NA	NA	Pleural thickening	Vimentin (+), CD31 (+), CD34 (−), UEA‐1 (+), VWF (+), Collagen IV (+)	None	Pleurectomy	NA
6		42/M	NA	NA	Pleural thickening and effusion	Vimentin (+), Keratin (+), CD34 (+), UEA‐1 (+), Collagen IV (+)	None	Pleurectomy	NA
7	Pinet et al.^2^	50/F	None	NA	Right pleural effusion	Factor VIII (+), BNH9 (−)	Peritoneum	Carboplatin, Etoposide	18 + (CR)
8–11	Crotty et al.^3^	55‐71/M	Chest pain (3), dyspnea (3), cough (1), fever (1), weight loss (1)	Asbestos, 3/4 (+), cigarette, 3/4 (+)	Right pleural effusion (4), pleural thickening or mass (1)	CD31 (+), CD34 (+), Factor VIII (+)	Lung (2) mediastinum lymph node (1), liver (1) retroperitoneal lymph node (1)	Decortication surgery	1–19 (mean = 10)
12	Zhang et al.^15^	66/M	NA	NA	Pleural effusion	Cytokeratin, 5/5 (+), CEA, 1/4 (+), CD31, 5/5 (+), CD34, 4/5 (+), factor VIII, 4/5 (+)	None	NA	6
13		45/M	NA	NA	Pleural effusion		None	NA	6
14		60/M	NA	NA	Bloody pleural effusion		None	NA	2
15		62/F	NA	NA	Pleural effusion, ascites		None	NA	1
16		53/M	NA	NA	Pleural effusion		None	NA	6
17	Attanoos et al.^20^	59/M	Pleuritic chest pain, dyspnea	Asbestos (+), cigarette (+)	Pleural effusion, diffuse pleural thickening	NA	None	NA	7
18		73/M	Chest infection, dyspnea	Asbestos (+), cigarette (+)	Pleural effusion, pleural fibrosis, and mass	NA	None	NA	9
19		33/M	Cough, pleuritic chest pain, dyspnea	Asbestos (+), cigarette (+)	Pleural effusion, pleural thickening	NA	Mediastinum, diaphragm	NA	8
20	Vitório et al.^21^	61/M	Chest pain, weight loss,	Asbestos (+), cigarette (+)	Pleural effusion, pleural thickening, small pleural nodule	CD31 (+). CD34 (+), Factor VIII (+)	None	Cisplatin, Etoposide	3
21	Al‐Shraim et al.^22^	51/M	Cough, dyspnea	NA	Left pleural effusion	CD31 (+), CD34 (+), Factor VIII (+)	Skin	INF‐α, decortication	24 +
22	Knuuttila et al.^23^	F/72	Dyspnea	NA	negative	CAM5.2 (−), vimentin (+), Calretinin (−), Muscle Actin (−), CD34 (−), S‐100 (−), CK56 (−) EMA (−)	None	NA	(113+)
23	Saqi et al.^24^	37/M	Dyspnea	Asbestos (−), cigarette (−)	Right‐sided pleural effusion, Pleural thickening	NA	None	Carboplatin, Etoposide, Avastin, decortication, thoracentesis	2
24	Lee YJ et al.^25^	31/F	Upper back and shoulder pain, chest pain	Asbestos (−), cigarette (−)	Nodular pleural thickening (in CT scan)	CD31 (+), CD34 (+), Factor VIII (+)	Lung, Bone	Radiation, Mesna‐Doxorubicin‐Ifosfamide‐Dacarbazine (MAID), Adriamycin	10
25	Martinez et al.^14^	37/F	Cough, dyspnea, back pain	NA	Pleural effusion	Vimentin (+), CD31 (+), CD34 (−)	None	Interferon, Thalidomide	NA
26	Liu et al.^26^	80/M	dyspnea, Right side chest pain	Asbestos (−), cigarette (−)	Right‐sided pleural effusion	CD31 (+), FLI1 (+), Pancytokeratin focal (+)	None	Chemotherapeutic, surgery	6
27	Andre et al.^27^	65/F	Chest pain	Asbestos (−), cigarette (−)	Pleural effusion	Ca125 (+)	None	Carboplatin, etoposide	6
28	Bocchino et al.^1^	58/F	Cough, dyspnea, chest pain	Asbestos (−), cigarette (−)	Pleural mass	CD31 (+), CD34 (+), factor VIII (+), ulex (+), Europeaus (+)	None	None	3
29	Kim et al.^28^	46/F	Right‐sided chest pain, cough	NA	Right‐sided pleural effusion	CD31 (+), CD34 (+)	Chest wall, Esophagus	Carboplatin, etoposide, Resection	23+
30	Márquez‐Medina et al.^16^	85/M	negative	NA	Right‐sided pleural effusion	CD31 (+), calretinin, (−) TTF‐1 (−), B72.3 (−), pankeratin (−), WT1 (−), CD34 (−), CD68 (−), p63 (−), 34betaE12 (−), CAM5.2 (−), S‐100 (−)	None	No treatment	7
31	Lazarus et al.^29^	42/M	Cough dyspnea chest pain	NA	Right pleural effusion	NA	Skin	Paclitaxel, bevacizumab	8
32		42/M	Fever, cough	Asbestos (−), cigarette (+)	Left pleural effusion	CD31 (+), CD34 (+)	None	Carboplatin, etoposide, bevacizumab	6
33	Chou et al.^30^	42/M	Chest pain, cough	NA	Pleural effusion, pleural thickness	CD31 (+), CD34 (+), Vimentin (+), AE1/AE3 (−), Calretinin (−)	Bone (thoracic spine)	Partial pleurectomy, radiotherapy, chemotherapy	14‐in progress
34		27/M	Cough, hoarseness, chest tenderness	NA	Pleural mass, left hemidiaphragm paralysis, pleural effusion	CD31 (+), CD34 (−), FLI‐1 (+), Vimentin (+), AE1/AE3 (−), Calretinin (−), S‐100 (−)	None	Radiotherapy, pleurectomy, Doxorubicin, cisplatin	7 ‐ CR
35	Bansal et al.^31^	51/F	left‐sided, non‐pleuritic chest pain, weight loss	NA	large loculated effusion with pleural thickening	CD31 (+), CD34 (+)	None	Doxorubicin	4
36	Photowala et al.^32^	NA	Hypoxemia, bilateral pleuretic chest pain, dyspnea	Asbestos (−), cigarette (−)	Small to moderate bilateral pleural effusion, Hemothorax	AE1/3 (+), CAM 5.2 (+), CD31 (+), calretinin (−), Cytokeratin‐5/6 (−), cytokeratin‐7 (−), cytokeratin‐20 (−), CD30 (−), CD117 (−), CD34 (−)	Pericardium	Thoracotomy	5,
37	Yu et al.^33^	39/F	NA	Asbestos (−), cigarette (−)	Left thoracic mass	NA	None	Carboplatin, etoposide	14‐ CR
38	Ha et al.^17^	71/M	Cough, dyspnea, fatigue, weight loss	Asbestos (−), cigarette (−)	Bilateral Pleural effusion, pleural and lung nodule,	CD31 (+), CD34 (−), CAMTA1 rearrangement (+)	None	NA	NA
39	Wethasinghe et al.^34^	41/M	Right lower chest pain, weight loss	Asbestos (−), cigarette (−)	pleural thickening	NA	None	radiotherapy	6 – In progress
40	Sanxi et al.^35^	40/M	Chest pain,	Asbestos (−), cigarette (+)	Left pleural effusion, Thickening of pleura	CD31 (+), CD34 (+), vimentin (+), AE1 / AE3 (+), Calretinin (−), Ki‐67 (<1% low), CK5 / 6 (−), FVIIRAg (−)	None	Gemcitabine, cisplatin	NA
41	Salijevska et al.^36^	36/F	Chest pain, dyspnea	Asbestos (−), cigarette (+)	complete opacification of the right hemothorax, right‐sided pleural effusion, pleural thickening	MNF116 (+), CK7 (+), D2‐40 (+), CD31 (+), CD34 (+)	lungs	Paclitaxel	6
42	Jamy et al.^9^	24/M	Abdominal pain, chest pain, dyspnea, nausea and vomiting, hemoptysis	NA	Pleural effusion, pleural thickening	CD31 (+); MOC‐31 (−), Ber‐Ep4 (−), p63 (−), OCT‐4 (−), alpha‐fetoprotein (−)	None	Carboplatin, paclitaxel	2
43	Kanemura et al.^15^	31/F	Dull back pain	NA	Pleural thickening, pleural effusion	AE1/AE3 (−), CAM5.2 (−), calretinin (−), WT‐1, CEA (−), TTF‐1 (−), D2‐40 (+), vimentin (+), CD34 (+), CD31 (+), factor VIII (+)	None	carboplatin, pemetrexed, Bevacizumab	6 ‐ CR
44	Fan et al.^37^	68/F	Back pain	Asbestos (−), cigarette (−)	Left pleural effusion and thickening	CD31 (+), M2a (+), carcinoembryonic antigen (D2‐40) (+), ERG (+), Ki67 (+), CD34 (−), CK7 (−), TTF‐1 (−), Napsin A (−), PCK (−), CK5/6 (−), WT‐1 (−), ALK D5f2 (−), E‐cadherin (−), Factor VIII (−)	None	NA	NA
45	Ferreiro et al^38^	85/M	Left pleuritic pain, progressive dyspnea, dry cough.	Asbestos (−), cigarette (+)	bilateral pleural effusion	Vimentin (+), cytokeratin (AE1–AE3) (+), CD31 (+), calretinin. (−) Ki‐67 (high)	None	Symptomatic	2
46	Albano et al.^39^	76/F	Chest pain, dyspnea, cough	NA	Pleural thickening, Right side pleural effusion	CD31(+), CD34 (+), panCK (−), AE1/AE3 (−), CK5/6 (−), EMA (−), calretinin (−), WT1 (−), TTF1 (−), Napsin (−)	None	Chest drainage	6
47	Rejeb et al.^37^	79/M	Chest pain, dyspnea, weight loss	NA	Pleural thickening, right side pleural effusion	CD34 (+)	None	Etoposide, Cisplatin	1
48	Takenaka et al.^40^	62/M	Right chest pain, dyspnea	Asbestos (−), cigarette (+)	Right side pleural effusion	Calretinin (−), CD31 (+), CD34 (+), CAMTA1 (+)	None	Extrapleural pneumonectomy, pazopanib	3.5
49	AlGhunaim et al.^41^	49/M	Cough, dyspnea, weight loss, chest pain	NA	Hemothorax	CD34 (+), CD31 (+), CK7 (−), CK20 (−), calretinin (−), TTF1 (−), napsin (−)	None	Chemotherapy	NA
50	Our case	68/M	Pleuritic chest pain, weight loss	Asbestos (−), cigarette (−)	Right side pleural effusion and mass	AE1.3 (+), CAM 5.2 (+), CD31 (+), ERG (+), CD34 (−), MUC4 (−), MOC31 (−), BerEp4 (−), TTF1 (−), calretinin (−), P40 (−), desmin (−), S100 (−), Melan‐A (−). KI69 elevated (70%).	None	Paclitaxel	8 ‐ In progress

Mallory initially described hemangioendothelioma (EH) in 1908, and later in 1943, Stout published a series of cases reporting a tumor of vascular origin from various sites that could be misdiagnosed as angiosarcoma.^6^, ^36^ In 1975, Dail and Leibow reported a tumor of intravascular bronchioloalveolar (IVBATT) which then in 1982 was named Epithelioid hemangioendothelioma (EHE) by Weiss and Enzinger, who reported twenty cases with gradually growing nodules of the lung with variant histology from malignant sarcomatous to benign granulomatous tumors.^4^, ^9^, ^15^, ^37^

A literature review of all EHE cases has demonstrated heterogeneity in its management and presentation. To our knowledge, this review summarizes by far the largest number of patients with pleural EHE. The average age of patients diagnosed with PEH was 53.72 (SD = 15.98) years, and 70% were males, which aligns with previous reports that pleural EHE commonly affects older men presenting with symptoms of the disease. In contrast, lung EHE affects middle‐aged and young women without symptoms^7^, ^8^, ^9^, ^37^, ^38^, ^39^ and cases in children are extremely rare.^40^ Among the cases, 8 (16%) had a history of asbestos exposure, while 6 (12%) had a history of cigarette smoking. Common clinical symptoms of pleural EHE include pleuritic chest pain (54%), dyspnea (46%), sputum production and cough (30%), and weight loss (16%). In comparison, less frequent symptoms included shoulder or back pain (8%), hemoptysis (6%), fever (4%), fatigue (4%), nausea vomiting (2%), night sweating (2%), hypoxemia (2%), and hoarseness (2%). Multiple uncommon symptoms have been reported as the presentation of pleural EHE, such as the rise of pigmented lesions on the patients back representing Leser–Trélat sign which happens to be a paraneoplastic syndrome from an unknown etiology, compression effect of the tumor on the myocardium in a middle‐age woman and spontaneous bilateral hemothorax indicating the vascular nature of this tumor.^20^, ^23^, ^24^ Amin et al. even reported a case of an accidental finding of EHE following a routine CXR due to gastroenteritis with pleural metastasis.^41^

Although the exact etiology of EHE has not been established, several theories have been reported to play a role in its pathogenesis, such as the use of contraceptives, exposure to radiation or asbestosis, occupation contaminants such as heavy industry, vinyl chloride inhalation, smoking, the role of biological substances in stimulation endothelial proliferation and angiogenesis and translocations in chromosomes (e.g., genetic disorders, *t* (1; 3) (p36.23; q25), WWTR1–CAMTA1 gene fusion, YAP1–TFE3 fusion).^2^, ^3^, ^9^, ^10^, ^25^, ^42^, ^43^, ^44^, ^45^, ^46^ It is worth mentioning that the patient in our report had no predisposing factors.

Even though EHE has an indolent nature, progression, metastasis, and recurrences can occur.^47^, ^48^, ^49^ The diagnosis of EHE is made upon histopathological evaluation and is characterized by solid nests of cells in a myxoid or hyaline stroma and tubulopapillary growth of short strands showing frequent intracytoplasmic vacuoles.^12^, ^50^, ^51^ In addition, vascular immunohistochemistry markers may be helpful in the confirmation of the diagnosis. CD31 had the highest positivity based on reviewed literature (32/32), followed by CD34 (23/31), Vimentin (11/11), Factor VIII (8/9), VWF (5/6), UEA1 (4/4), collagen IV (4/4), Ki67 (3/4), FLI1 (2/2), CAM52 (2/4), Keratin (7/11), and AE1/AE3 (4/7). Factors that contribute to the tumor's aggressiveness include necrosis, an increase in cellularity, and a high mitotic rate which, in this case, can be labeled as malignant EHE.^47^, ^50^

Various malignancies can involve pleura; thus, the differential diagnosis of angiosarcomas, mesothelioma, adenocarcinoma, and metastatic neoplasms is essential to distinguish the exact pathology from another.^3^, ^5^, ^12^, ^15^, ^49^ Mesothelial immunohistochemical markers such as WT1 and calretinin (0/11 in our study) can help segregate pleural EHE from mesothelioma. Lastly, distinguishing pleural EHE from angiosarcomas is vital since most angiosarcomas are fatal.^52^

Based on our review, pleural effusion and pleural thickening (nodular or smooth) have been the typical manifestation of pleural EHE in radiological examination (76% and 32%, respectively). However, these findings should have been considered nonspecific due to their similarity to radiological findings in mesothelioma and diffuse pleural carcinomatosis.^7^, ^9^, ^39^, ^49^ Fibrosis, mediastinal lymphadenopathy, hemidiaphragm paralysis, hemothorax, and invasion of the diaphragm, ribs, and vertebrae were uncommon findings among pleural EH cases.^9^, ^10^, ^11^ As our case had an increased 18‐fluorine‐fluorodeoxyglucose (18F‐FDG) activity, two reports also showed an increase in uptake of FDG in their cases, indicating the aggressiveness of the tumor and its rapidly growing feature. It can also detect other tumor foci in other parts of the body and as a marker for decision‐making in therapeutic approaches.^53^, ^54^ Malignant tumor cells often show higher FDG uptake, and SUV ≥2.5 is considered to be a criterion for malignancy (specificity, sensitivity, and accuracy of 82%, 97%, and 92%, respectively).^54^

In literature, no standard treatment has been established for pleural EHE, yet, some studies used surgery (11: 22%), chemotherapy and radiotherapy (18: 36%), combination therapy (6: 12%), and some only symptomatic therapy (5: 10%). Surgical approaches are mostly considered when the tumor is local; nevertheless, microscopic tumors may remain in the chest wall when done without chemotherapy.^15^, ^24^, ^34^ A combination of chemotherapy with surgical treatment, especially administering etoposide and carboplatin, has been effective in reports that led to a disease‐free period of more than 14 months.^19^, ^24^ Also, the use of six cycles of carboplatin and etoposide had resulted in the complete remission of the disease. In contrast, in another case, the combination use of etoposide and cisplatin resulted in only limited response in another case.^2^, ^4^ In 2008, Lee et al. reported a 31‐year‐old woman diagnosed with pleural EHE with extension to the bone and lung, which was treated with Doxorubicin, ifosfamide, mesna, and dacarbazine (MAID) and palliative radiotherapy on the spine which led to 10 months survival of the patient.^15^ Also, a report demonstrated no benefit in administering Bevacizumab, an anti‐vascular endothelial growth factor monoclonal antibody, plus the use of etoposide and carboplatin.

After a period of observation, surgical resection is the treatment of choice in cases with proven unifocal disease or with technically resectable locoregional metastases. Pleural stripping or pneumonectomy may be considered individually, taking into account the severity of the disease and the expected benefit. Local ablative techniques may be considered when EHE involvement is limited to the lungs.^55^

Despite the lack of a standard medical approach, patients with metastatic disease and unequivocal evidence of disease progression, worsening symptoms, or organ dysfunction are candidates for systemic treatment. Conventional soft tissue sarcomas chemotherapy appears to have very limited activity and should be reserved for cases that are more aggressive or rapidly progressing, such as high‐grade soft tissue sarcomas.^56^ Therefore, further data regarding patients in clinical trials are required to draw a clear conclusion.

Interferon,^57^, ^58^ thalidomide,^59^ multi‐tyrosine kinase inhibitors (usually with a strong vascular endothelial growth factor receptor [VEGFR] inhibitory property),^60^, ^61^, ^62^, ^63^, ^64^, ^65^, ^66^ and mechanistic target of rapamycin (mTOR) inhibitors^59^, ^67^, ^68^, ^69^, ^70^ have all shown antitumor activity retrospectively. In our report, the patient was initially treated with pazopanib, which inhibits tumors growth by inhibiting angiogenesis via inhibiting cell surface VEGFR (VEGFR‐1, VEGFR‐2, VEGFR‐3), platelet‐derived growth factor receptors (PDGFR‐alpha and ‐beta), fibroblast growth factor receptor (FGFR‐1 and ‐3), cytokine receptor (cKIT), interleukin‐2 receptor inducible T‐cell kinase, lymphocyte‐specific protein tyrosine kinase (Lck), and transmembrane glycoprotein receptor tyrosine kinase (c‐Fms).^71^ Based on previous reports, mTOR inhibitors have shown the most clinical activity, with progression‐free survival and overall survival in 1 to 2 years, respectively, and approximately 10% of patients have even longer progression‐free survival.^55^

In reviewed pleural EHE cases, out of 50 cases, 40 (80%) patients died, 4 had complete remission, and six are still pending, which demonstrated the disease's poor prognosis. The results of our review demonstrate an average of 9.98 months of disease course. In literature, the prognosis of EHE is variable depending on the origin site. Pulmonary EHE has been reported to have a better prognosis than pleural EHE despite the extensive involvement of parenchymal tissue with a long life expectancy.^37^, ^38^ According to reports, an indicator for poor prognosis includes being symptomatic at presentation, metastasis, peripheral lymphadenopathy, and lymphangitic tumor spread, extensive interstitial or intravascular tumor spread, fibrous pleuritis with an extrapleural proliferation of tumor cells, pleural effusion, and spindle tumor cells.^37^, ^38^, ^39^, ^41^, ^48^

In conclusion, based on our review, although rare, taking into consideration the diagnosis of pleural EHE is essential due to the disease's high mortality. Further research and reporting of cases are necessary to add to the information about the clinical progression and the natural history of this rare malignancy to provide a therapeutic approach to the disease.

## AUTHOR CONTRIBUTIONS

AR designed the study. RS collected the data and performed a review of the literature. AE and KR drafted the manuscript. All authors proofread and accepted the final version of the manuscript.

## FUNDING INFORMATION

No financial support was received for this report.

## CONFLICT OF INTEREST

The authors declare that they have no competing interests.

## ETHICAL APPROVAL

Written informed consent was obtained from the patient in our study. The purpose of this research was thoroughly explained to the patient, and he was assured that their information would be kept confidential by the researcher. The present study was approved by the Medical Ethics Committee of the academy.

## CONSENT

Written informed consent was obtained from the patient for publication of this case report and any accompanying images. A copy of the written consent is available for review by the editor‐in‐chief of this journal.

## Data Availability

SPSS data of the participant can be requested from the authors. Please write to the corresponding author if you are interested in such data.
